# Sensory-Neural Hearing Loss as an Early Rebound Relapse after Fingolimod Cessation in Multiple Sclerosis 

**DOI:** 10.22038/ijorl.2020.40858.2334

**Published:** 2020-07

**Authors:** Abdoreza Naser Moghadasi, Maryam Poursadeghfard, Tayebeh Kazemi, Samaneh Hosseini

**Affiliations:** 1 *Multiple Sclerosis Research Center, Neuroscience Institute, Tehran University of Medical Sciences, Tehran, Iran.*; 2 *Clinical Neurology Research Center, Shiraz University of Medical Sciences, Shiraz, Iran.*; 3 *Otolaryngology Research Center, Department of Otolaryngology, Shiraz University of Medical Sciences, Shiraz, Iran.*; 4 *Neurosciences Research Center, Tabriz University of Medical Sciences, Tabriz, Iran.*

**Keywords:** Fingolimod, Multiple sclerosis, Rebound activity

## Abstract

**Introduction:**

Multiple sclerosis (MS) is a lifelong disease of the brain and spinal cord. Fingolimod is an oral drug which modulates the S1P receptor and is used for relapsing remitting form of MS and can causes rebound activity if it is ceased even in a short period of washout time.

**Case Report:**

Here, we introduce a young girl, a known case of MS, who developed reversible isolated unilateral sensory-neural hearing loss along imaging activity two weeks after stopping fingolimod. The patient responded well to the intravenous corticosteroid therapy which is the first line treatment of new MS attack.

**Conclusion:**

fingolimod cessation can cause rebound activity in a short period of the time. It is important to consider any new neurological sign and symptom as a rebound activity during washout time. Although SNHL is not common in MS, it could be presented as an unusual manifestation of rebound relapse after stopping fingolimod.

## Introduction

Multiple sclerosis (MS) is a lifelong disease of the brain and spinal cord which is presented by scattered areas of both demyelination and axonal damage. The disease is distributed throughout the central nervous system (CNS), resulting in different neurological presentations (1). The disease may be mono- or poly-symptomatic and the most common presentations are optic neuritis, cerebellum, brain stem and spinal cord and sometimes cortical cerebral manifestations (2). 

Cranial nerves involvement either in combination with other parts of the brain stem or in isolated form as a presenting or exacerbating sign of MS has been previously more and less reported. In a study in 2008, the most prevalent affected cranial nerve after the optic verve was the trigeminal nerve followed by the facial, abducens, oculomotor and cochlear nerves. Only about 50% of the patients had imaging findings which could clarify the symptoms (3). 

Though otology symptoms arise from either cochlear nerve root exit zone or more proximal portions of the auditory pathway, they are less common than other cranial nerves and brain stem manifestations in MS; recent data indicate the auditory involvement among different stages of the disease, as a presenting symptom or a new attack in a previously established disease (4). 

Currently, different oral and injectable disease modifying drugs (DMDs) are used for MS. Fingolimod is an oral DMD which modulates the S1P receptor and is used for relapsing remitting form of MS. It is a prodrug and could be phosphorylated to fingolimod-p in a reversible manner. Fingolimod-p binds to 4 of 5 sphingosine 1-phosphate receptor (S1PR) subtypes: S1PR1, S1PR3, S1PR4, S1PR5, but not S1PR2 (5).

Fingolimod-p is an agonist as well as functional antagonist for S1PR1 and leading to a pharmacological null state (6). Fingolimod causes down-modulation of S1PR1 expression on lymphocytes and renders them not to egress from lymph nodes and they rapidly reduce in thoracic duct, peripheral blood and spleen (7,8). The exact mechanism of fingolimod in MS is unknown, but probably inhibition of lymphocyte egression from lymph nodes interfere with potentially autoreactive lymphocytes trafficking to CNS. It can cause rebound activity if it is ceased even in a short period of washout time (9). Rebound activity is defined severe reactivation of the disease with the high level of the MS activity compared with the patient’s pre-medication state. It is taught to occur due to over-expression of S1P1 on the entrapped lymphocytes causing migration to the CNS and induction of the inflammation (10). To the best of our knowledge, there is no report of hearing loss as fingolimod rebound activity in MS patients. Here, we introduce a young girl, a known case of MS, who developed isolated unilateral sensory-neural hearing loss (SNHL) 2 weeks after stopping fingolimod.

## Case Report

**Fig 1:**
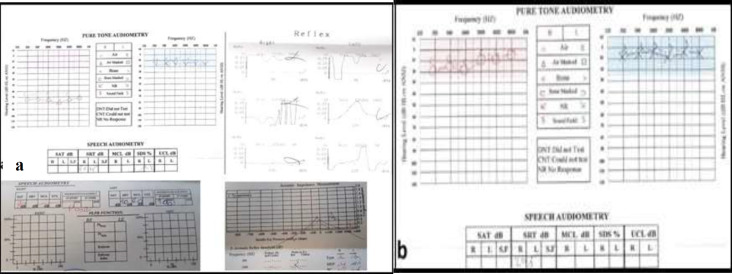
The results of the audiometric test of both sides; (**a**)-before treatment, pure tone audiometry of the right ear shows a decrease in both air and bone conduction indicative of SNHL. Speech audiometry in the right side demonstrates a significant decrease in speech reception test and poor quality of speech reception score (**b**)-after treatmen, so a retro-cochlear damage of the auditory pathway was considered

**Fig 2:**
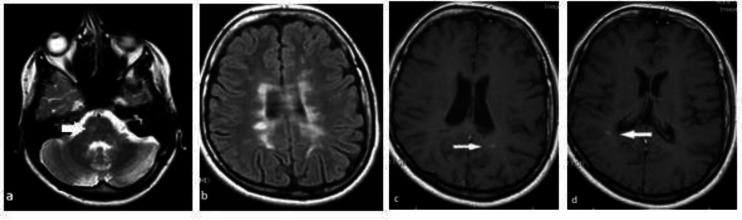
Axial views of the T2 weighted and FLAIR (a and b) and T1 weighted imaging with contrast (c and d) of the brain MRI. A new T2 lesion in the right side of the pons (thick white arrow) and 2 gadolinium enhancing lesions (thin white arrows) in the both sides of the centrum semiovale are seen

The patient is a 19-year-old girl, a known case of RRMS starting from 5 years ago. She was well until the time of the MS diagnosis, about 5 years ago. With the disease diagnosis, subcutaneous interferon beta 1-a was started three times a week for the first 3 years of her disease and after that switched to fingolimod 0.5 milligram daily owing to new clinical and radiological relapse. She was stable during fingolimod consumption without disease activity (clinical and imaging aspects). However; she was tiered of taking medicine and discontinued her drug on her own. About 2 weeks after cessation of fingolimod, she developed acute onset of hearing loss and tinnitus in her right ear after cessation of fingolimod for 2 weeks. There was no history of vertigo, headache or ear pain, recent trauma to the head, otitis media, upper respiratory or other viral infections, and family history of hearing loss. She also did not use new medication with ototoxicity side effect in the previous weeks. She was on the MS DMDs and was well since the time of the MS diagnosis, about 5 years ago, until 2 weeks before the onset of the hearing loss (subcutaneous interferon 1a three times a week for 3 years and then daily oral fingolimod because of new clinical and radiological relapse). General and neurological examination, especially of the head and neck parts, was not significant except for a decrease in hearing in the right ear at the time of the hospital admission. On otoscopic evaluation, she had intact bilateral tympanic membranes. After consulting with otolaryngologist and completing audiometric examination and work-up, SNHL was diagnosed. The results of the audiometric tests before and after treatment are demonstrated in (Fig.1(a,b)). Routine laboratory tests, autoimmune profiles and vasculitis markers all showed to be normal. New brain MRI showed one new T2 lesion in the right side of the pons and 2 small gad enhancing lesions in the supratentorial area (Fig. 2).

Thus, with impression of probable new MS attack resulting from fingolimod cessation due to both clinical and radiological activity, she received 5 grams of intravenous (IV) methyl prednisolone and improved significantly with the pulse of the corticosteroid therapy.

## Discussion

SNHL is usually described as loss of hearing about 30dB or more in at least 3 sequential frequencies of pure tone audiometry within 3 days. In the majority of the cases, the etiology is unidentified. Among well-known causes, infections, metabolic disorders, neoplastic diseases and ultimately many autoimmune diseases are pointed (11). Association of sudden SNHL with vestibular symptom is highly indicative for labyrinthitis (toxic or bacterial) which is a peripheral vestibular disorder. Our case did not complain of vertigo, disequilibrium, tinnitus or ear pain. 

Idiopathic sudden onset SNHL is a by exclusion diagnosis.  In the setting of this case who suffered from multiple sclerosis and was on medical therapy which was discontinued recently, it seems that we could not classified her hearing loss as idiopathic type, although we cannot rule out it exclusively.

 Basic audiologic tests also can provide essential diagnostic clues as to whether SNHL is cochlear or retro-cochlear in origin. We should have a high degree of suspicion for retro-cochlear etiologies when SNHL is asymmetric, speech discrimination is abnormally reduced or asymmetric, or abnormalities in acoustic reflex are apparent in audiometry.

MS is one of those types of autoimmune disease in which symptoms could be raised from different parts of the CNS; however, some parts were involved less frequently. Our patient had MS for 5 years as an underlying autoimmune disorder; while all other autoimmune panel were negative. Indeed, she had no history of infection, trauma, and all new laboratory tests were not significant.

Cochlear division of the 8^th^ cranial verve (vestibulocochlear nerve) or its central pathway is not a common site of presentation in MS, but few recent data are indicating of more MS patients with SNHL than formerly was thought. The first cases about the relationship between SNHL and MS were reported in 1950s and 1960s (12). After that, other reports about association of SNHL to MS were published. Tekin and colleague reported a 30-year-old lady with the new attack of MS who got into SNHL 3.5 years after the diagnosis of MS. Like our case, their patient also recovered after a while with mild residual symptoms and without recurrence in the year after (13). In another report in 2006, a 15-year-old girl was introduced as a new case diagnosed with MS after evaluation for recent SNHL which was actually the presenting symptom of MS. This patient also completely improved after 2 months (14). In a systematic review in 2018, 72% of 1533 MS patients with SNHL were female and most of them had relapsing-remitting form of MS. In addition, the majority of the cases presented with sudden onset of SNHL, especially in the early stage of the disease, whereas progressive form occurred in the later stage of MS (15). 

Data about MS and SNHL is varied and sometimes opposite. For example, in a collected data reported from 2004 to 2014, 0.7% of all MS patients were diagnosed with sudden SNHL during their diseases. The authors concluded that sudden SNHL was a rare symptom of MS. In this study, women were dominant and the mean time from MS diagnosis to the occurrence of SNHL was 9.3 years (12). In contrast, in another survey from 2004 to 2009, 4.35% of MS patients developed SNHL and the authors stated that hearing loss was not uncommon in MS (16). A rate of 4-10% of SNHL during relapse or remission of MS is also reported in the literature (17).

What is common in all the above reports is that SNHL is more common in females than males with MS and in most cases, no identified plaque was detected. In addition, complete (and sometimes incomplete) recovery happens and it is supposed that hearing loss in MS would be temporary and returnable; actually, it has a good prognosis. Hence, it could be assumed as a new attack of the disease responding to the corticosteroid. 

Nevertheless, in none of the above reports, rebound activity was described. There are reports about rebound syndrome after fingolimod discontinuation which usually happens between 4 weeks to 4 months (9), but as far as we searched, no isolated SNHL was previously reported in the literature in these patients.

The girl presented here developed new symptoms in a very short time after withdrawal from the drug although she had a very good outcome with complete clinical recovery after IV steroid. Her new imaging showed a new T2 weighted lesion in the right side of the pons which supports the idea of upper auditory involvement in MS and 2 gadolinium enhancing lesions in the centrum semiovale which suggested new disease activity. 

Different parts of the auditory pathways (cochlea, cochlear nerve, brain stem and cortex) were cited as a cause of hearing loss in MS (15). Because she had no any symptoms of vestibular portion of the cranial nerve 8, the localization of the underlying pathology is estimated to be more proximal part of the auditory pathway. Nevertheless, she did not have any other manifestations of the brain stem and it could be interpreted that low-resolution MRI (3T) is sometimes unable to detect the MS plaque in the nucleus or nerve root exit zone. 

## Conclusion

Fingolimod cessation can cause rebound activity in a short period of the time. It is important to consider any new neurological sign and symptom as a rebound activity during washout time. Although SNHL is not common in MS, it could be presented as an unusual manifestation of rebound relapse after stopping fingolimod. 
